# Distinct variation in taste quality of Congou black tea during a single spring season

**DOI:** 10.1002/fsn3.1467

**Published:** 2020-02-18

**Authors:** Penghui Yu, Hao Huang, Xi Zhao, Ni Zhong, Hongfa Zheng, Yushun Gong

**Affiliations:** ^1^ Key Laboratory of Tea Science of Ministry of Education Hunan Agricultural University Changsha China; ^2^ Tea Research Institute of Hunan Academy of Agricultural Sciences Changsha China; ^3^ National Research Center of Engineering Technology for Utilization of Functional Ingredients from Botanicals Hunan Agricultural University Changsha China

**Keywords:** black tea, electronic tongue, sensory evaluation, spring season, taste quality

## Abstract

The quality of Congou black tea fluctuates greatly with the changing seasons. However, variations in the taste quality of Congou black tea manufactured during a single spring season are far from clear. Here, we analyzed the taste quality of HuangJinCha (HJC) Congou black tea using sensory evaluation and found the taste quality of black tea manufactured in the early spring was better than that manufactured in the late spring. Principal component analysis (PCA) and cluster analysis for the data from the electronic tongue confirmed the variation and revealed that April 4 may be the critical time point at which variations in taste quality become apparent. The contents of tea polyphenols (TP), total catechins (TC), total flavones, (‐)‐epigallocatechin gallate (EGCG), (‐)‐epicatechin gallate (ECG), and gallic acid (GA) showed increasing trends, whereas total amino acids (TAA) declined over time. Moreover, the variations in total amino acids (r = 0.846) and total flavones (r = ‐ 0.858) were highly significantly correlated with the average taste quality score (*p* < .01), suggesting these compounds were the primary factors responsible for the fluctuation in taste quality of Congou black tea processed during a single spring season.

## Graphical abstract

The taste quality of Huangjincha Congou black tea manufactured in the early spring was better than that manufactured in the late spring. The variations in total amino acids and total flavones were significantly correlated with the average taste quality score (P < 0.01), suggesting these two compounds were the primary factors responsible for the fluctuation in taste quality.

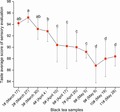

## 1 INTRODUCTION

Tea is one of the most popular drinks worldwide, and 78% is consumed as black tea due to its health benefits and satisfactory sensory characteristics. Fully fermented black tea contains higher levels of oxidation products (including dimeric and polymeric catechins) than unfermented green tea. In recent years, it has been reported that black tea possesses potential protective effects against cancer, cardiovascular diseases, chronic inflammatory diseases, metabolic syndrome, and neurodegenerative diseases (Pan et al., 2013) and also regulates several cellular stressors including oxidative, hypertonic, osmosis, heat, and UV irradiation (Xiong et al., 2014; Yuan et al., 2018). Taste quality after tea brewing is one of the most important factors affecting consumers' preference. Congou black tea manufactured in the spring has a lower level of bitterness and astringency than teas manufactured during summer months, catering to the satisfaction of numerous consumers, especially in Asian countries.

The taste quality of tea is influenced by many factors such as variety, growth environment (climate, soil, and altitude), horticultural practices, harvest seasons, and manufacturing methods (Ahmed et al., 2014; Jayasekera, Kaur, Molan, Garg, & Moughan, 2014; Wang, Wei, et al., 2011b). In China, most tea leaves in the spring are plucked and manufactured into green tea because of the higher content of amino acids; whereas most tea leaves in summer and autumn are plucked and manufactured into black tea for the higher contents of polyphenols. Furthermore, the specific compounds present in tea leaves differ seasonally and thus, may significantly influence taste quality. The taste quality of green tea manufactured during different plucking seasons has been widely reported (Dai et al., 2015; Liu et al., 2016; Xu, Song, Li, & Wan, 2012); however, few studies have described the seasonal variation on taste quality of Congou black tea, especially the variation that occurs within a single growing season. In fact, the taste quality of Congou black tea is ascribed not only to the distinct compounds in preharvest leaves from different time points, but also to the activity of enzymes (including polyphenol oxidase and peroxidase) during postharvest processing. The degree of polyphenol oxidase (PPO) and peroxidase (POD) activities depends on the variety of tea trees as well as on seasonal climatic conditions which significantly influence the full conversion of polyphenols and the formation of theaflavins (TFs) and thearubigins (TRs). Thus, we hypothesized that the taste quality of black tea may vary greatly even within a single spring season.

In this study, we developed a sensory evaluation method combined with electronic tongue analysis to determine whether Congou black tea samples manufactured during different periods of the spring season possessed different taste quality. After quantifying the contents of the main taste components in black tea samples, we correlated the variation of contents with taste quality scores and suggested several compounds responsible for the spring seasonal variation in taste quality.

## 2 MATERIALS AND METHODS

### 2.1 Chemicals

(‐)‐Catechin (C), (‐)‐epigallocatechin (EGC), (‐)‐gallocatechin (GC), (‐)‐epicatechin (EC), (‐)‐epicatechin gallate (ECG), (‐)‐epigallocatechin gallate (EGCG), (‐)‐gallocatechin gallate (GCG), anhydrous glucose, caffeine (CAF), and gallic acid (GA) were purchased from Shanghai Yuanye Biotechnology Co., Ltd. Company (Shanghai, China). Alanine (ala), arginine (arg), aspartic acid (asp), cysteine (cys), glutamic acid (glu), glycine (gly), histidine (his), isoleucine (ile), leucine (leu), methionine (met), phenylalanine (phe), proline (pro), serine (ser), threonine (thr), tryptophan (trp), tyrosine (tyr), valine (val), theanine, the AccQ‐Fluor Reagent Kit, and AccQ‐Tag eluent were purchased from BDH Chemicals Ltd. (Poole, UK). Acetonitrile (chromatographically pure), sodium carbonate, methanol (chromatographically pure), concentrated sulfuric acid, anthrone, ferrous tartrate (analytically pure), disodium hydrogen phosphate, and potassium dihydrogen phosphate were purchased from Sinopharm Chemical Reagent Co., Ltd. (Shanghai, China). Pure water from China Resources C'estbon Beverage Co., Ltd. (Shenzhen, China) was used throughout the study.

### 2.2 Tea sample treatments

Fresh tea leaves of the HJC variety were harvested from the same tea garden of Dai Linbing Tea Professional Cooperative (109.8 E, 28.5 N, Baojing, Hunan, China) in the early spring (March, average temperature 14.1°C), mid spring (April, average temperature 19.1°C), and late spring (May, average temperature 22.9°C) in 2018. Tea leaves were plucked with the same standard of one bud and one leaf in the sunny day (unequal intervals) and manufactured using the same method for Congou black tea. Briefly, after approximately 12 hr of withering at 26°C and 70% relative humidity, a rolling machine (Sunyoung Machinery Ltd., Zhejiang, China) rolled the tea leaves for 70 min. Then, fermentation occurred in an environmentally controlled cabinet at 30°C and 90% relative humidity for 4 hr. In the drying stage, tea leaves were initially roasted at 120°C for 15 min and then roasted to a moisture of approximately 7% at 90°C for 60 min in an aroma‐enhancing machine. Black tea samples were ground into powder (100 mesh) using a tube mill and stored at 4°C for future use.

### 2.3 Sensory evaluations

Sensory evaluation was performed according to China National Standards outlined in GB/T 23776–2018. Briefly, black tea samples were double‐blinded before 3 g tea was brewed using 150 ml of boiling water for 5 min prior to filtration. Black tea infusions were scored at room temperature (approximately 25°C) by a professional panel composed of seven specialists (four men and three women, 29–45 years old). Each specialist had a minimum of 150 hr of tea sensory evaluation experience from the Tea Research Institute of Hunan Academy of Agricultural Sciences. The sensory response was evaluated on a 100‐point scale where 90–99 indicated high mellow and fresh, 80–89 indicated neutral mellow and fresh, and 70–79 indicated low mellow and fresh.

### 2.4 Electronic tongue measurements

Experiments were performed using an α‐ASTREE electronic tongue system (Alpha MOS, Toulouse, France) equipped with an Ag/AgCl reference electrode and 7 taste sensors (SRS, SWS, BRS, STS, UMS, GPS, and SPS, representing sour, sweet, bitter, salty, umami, and two combination tastes, respectively). The methods for electronic tongue measurement were based on those described in a previous study (Xiao & Wang, 2009). Congou black tea (3 g) was infused with 150 ml of deionized boiled water for 5 min, and then the leaves were removed. Each tea sample was infused 3 times and cooled to 25 ± 2°C, and 100 ml infusion of each sample was used in the measurement. Before detecting the infusion, the electronic tongue system was self‐tested, sensor activated, calibrated, and diagnosed successively. The data‐collection time was 120 s, and stable data from 110 s to 120 s were taken as the output value. A computer recorded the response of the e‐tongue every second. Each tea sample infusion was measured 7 times repeatedly, and the last 3 stable data points were selected for analysis.

### 2.5 Chemical analyses

#### 2.5.1 Tea polyphenols, total flavones, soluble sugar, theaflavins, thearubigins, and theabrownins determination

The determination of tea polyphenols (TP), total flavones, soluble sugar, theaflavins, thearubigins, and theabrownins was based on the methods described below (Turkmen, Sari, & Velioglu, 2006). Determination of TP was accomplished using ferrous tartrate spectrophotometry (Turkmen et al., 2006). Determination of total flavones used the aluminum trichloride colorimetric method (Karimi, Moradi, Alidadi, & Hashemi, 2016). Determination of tea soluble sugar (SS) used the fluorenone colorimetric method (Xu, Liu, et al., 2018a). Determination of TFs, TRs, and theabrownins (TBs) used the system analysis method (Jiang, Hua, Wang, Yuan, & Ma, 2018).

#### 2.5.2 Analyses of catechins, caffeine, and gallic acid

The composition of TC, CAF, and GA in the extract was determined with an HPLC system (LC‐2010AHT, Shimadzu Corp) equipped with a Shim‐pack VP‐ODS column (5 μm, 150 mm × 4.6 mm id, 35°C) at 278 nm according to the methodology described in a previous study (Wang, Liu, et al., 2011a). Black tea powder (3 g) was infused with 500 ml boiling deionized water and maintained for 45 min prior to filtering with a 0.45 µm membrane. Solvents A (water) and B (N, N‐dimethylformamide: methanol: acetic acid, 20:1:0.5) were run in linear gradients with B increasing from 14% to 23% over 13 min, from 23% to 36% for the next 12 min, and maintained for 3 min thereafter at a rate of 1.0 ml/min. Catechins, GA, and CAF were identified by comparison of retention times and spectra of standard solutions. Analytes were quantified using external calibration standards.

#### 2.5.3 Analyses of free amino acids

The composition of free amino acids was measured using the AccQ‐Fluor Reagent Kit according to the manufacturer's specifications. Separation was performed on the HPLC system equipped with a Waters AccQ‐Tag reversed‐phase HPLC column (150 mm × 3.9 mm, 5 µm), according to the manufacturer's specifications with slight modifications. Black tea powder (3 g) was infused with 500 ml boiling deionized water and maintained for 45 min prior to filtration with a 0.45 µm membrane. Amino acid standard solutions were dissolved in 0.1 mol/L HCl and filtered through a membrane for further use. Mobile phase A consisted of AccQ‐Tag Eluent A Concentrate in deionized water (1:10 v/v) and mobile phase B consisted of 60% acetonitrile in deionized water. The sample injection volume was 10 µl, and the flow rate was 1.0 ml/min. Column temperature was set at 37°C. Amino acids were detected at 248 nm and identified by comparison of retention times and spectra of standard solutions. Quantification was done via external calibration curves.

### 2.6 Data processing

The data were expressed as mean + standard deviation (*SD*). Principal component analysis was performed using Simca‐p 14 software (Umetrics AB, Umeä, Sweden). Heat map analysis and cluster analysis were performed with Origin software (version 2017, USA). The significance level was calculated by one‐way analysis of variance (ANOVA) using IBM SPSS statistics software (version 24.0, USA). The statistical significance is indicated in the figures (* *p* < .05, ** *p* < .01).

## 3 RESULTS

### 3.1 Taste quality of black tea decreased in sensory evaluations with each passing month during spring time

**Figure 1:**
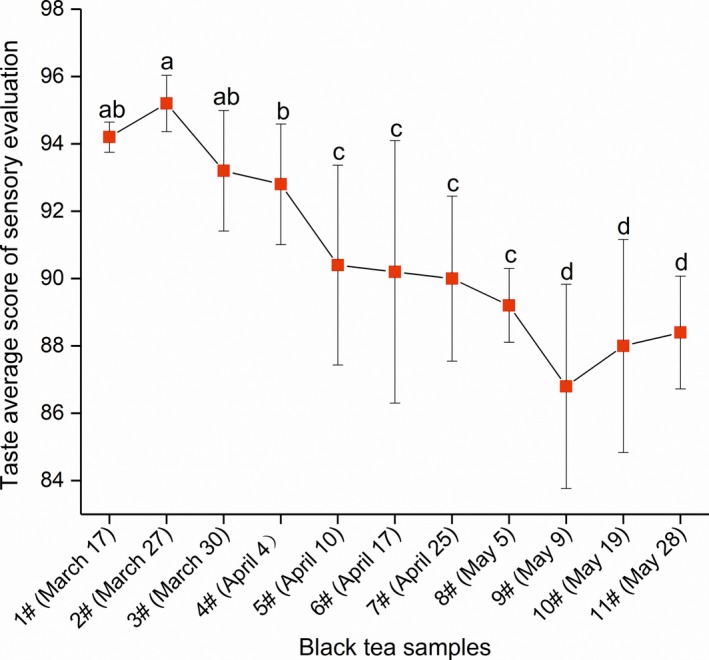
Sensory evaluation of black tea taste during a single spring season. Different lowercase letters in the figure indicate significant (*p* < .05) differences in the taste average score of black tea samples

Previous studies focused on the taste quality variation of Congou black tea manufactured in different seasons. In this study, the taste quality of black tea manufactured during a single spring season was investigated. The average score of taste quality generally decreased with the progression of time (Figure 1). Black tea manufactured in the early spring tasted fresher and mellower than that manufactured in the late spring. The average score on taste quality of tea samples decreased significantly from 95.2 in tea sample 2 to 86.4 in tea sample 9 (*p* < .05). The results suggested that the taste of HJC Congou black tea during different periods of spring can be divided into several types, and the tea samples from early spring had a relatively better taste quality.

### 3.2 Analysis from an electronic tongue confirmed the variation in black tea taste quality

**Figure 2:**
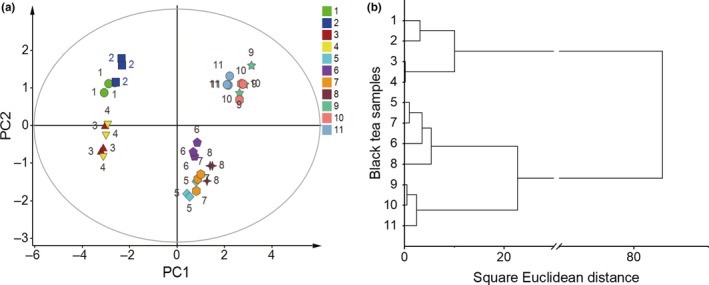
Electronic tongue analysis of HuangJinCha (HJC) black tea samples: (a) Principal component analysis (PCA) score plot; (b) Cluster analysis

To verify the sensory evaluation results, we used the data from an electronic tongue to distinguish the taste quality of HJC black tea manufactured throughout the spring. To confirm that the electronic tongue sensors possessed a reliable repeatability for the tea infusion, we calculated the relative standard deviation (RSD) of the sensors' response value and found all levels were less than 1.9% (Figure S1). The PCA of the electronic tongue sensor response value showed that the front two principal components reflected 77.86% and 18.48% of the original information in the tea sample, indicating that the taste quality of black tea can be assessed using the PCA model (Table S1). As depicted in Figure 2a, taste quality during different periods of spring can be divided into four types according to the relative distance, which represents the difference in taste. Black tea samples 1 and 2, 3 and 4, 5 to 8, and 9 to 11 were grouped together. Interestingly, black tea samples 1 to 4 (before April 4) and 5 to 11 (after April 4) were separated by principal component 1 (PC 1) and reflected the most information about the tea quality (Figure 2a), implying that April 4 may be the critical time point in the difference for black tea taste quality. In addition, we also investigated the different sensor taste scores from the electronic tongue through cluster analysis (Figure 2b). When the average Euclidean distance was 10, the tea samples could be clearly divided into 4 categories (tea samples 1 and 2, 3 and 4, 5 to 8, and 9 to 11). Moreover, tea samples 1 to 4 and 5 to 11 were grouped together, suggesting a great gap in taste quality between these two sets of tea samples. The results of cluster analysis were consistent with the results of PCA and the sensory evaluation.

### 3.3 Chemical compounds of black tea manufactured during a single spring season differed in content

**Figure 3:**
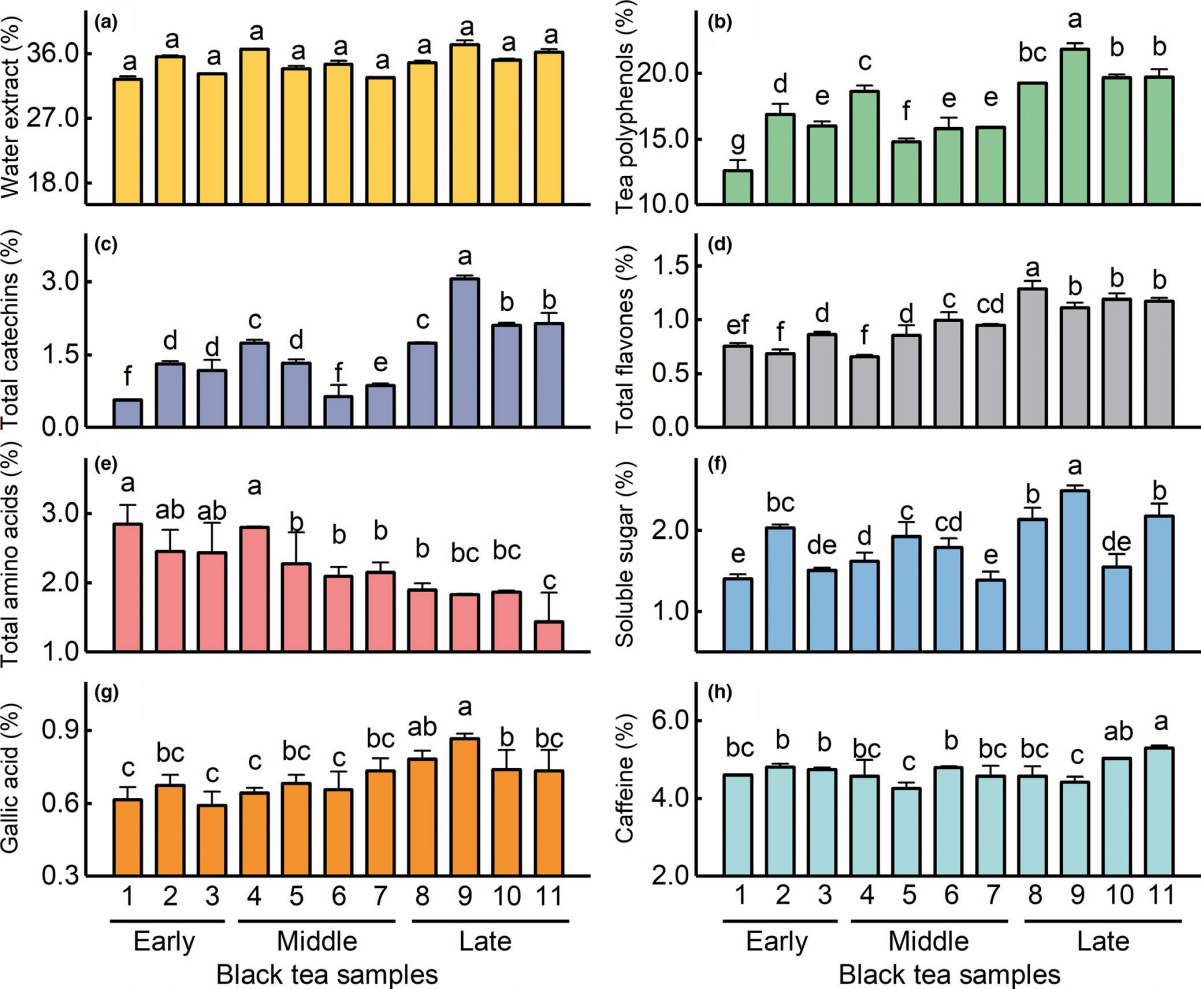
Contents of chemical compounds in black tea samples manufactured during a single spring. (a) Water extract. (b) Tea polyphenols. (c) Total catechins. (d) Total flavones. (e) Total amino acids. (f) Soluble sugar. (g) Gallic acid. (h) Caffeine. Different lowercase letters in the figure indicate significant (*p* < .05) differences in the contents of compounds

To understand the variation on tea taste quality in spring, we further analyzed the contents of the chemical compounds. As shown in Figure 3, the content of most taste compounds changed significantly (*p* < .05) in black tea samples save for water extract (WE). The TP, TC, total flavones, and GA content exhibited similar increasing trends, whereas the content of TAA showed a decreasing trend (Figure 3b‐f). However, neither the content of SS (Figure 3g) or CAF (Figure 3h) exhibited obvious changes in trends. Based on our findings, we speculated that the free amino acids and tea polyphenols (including catechins, flavones, and GA) may play an important role in the tea taste quality.

**Figure 4:**
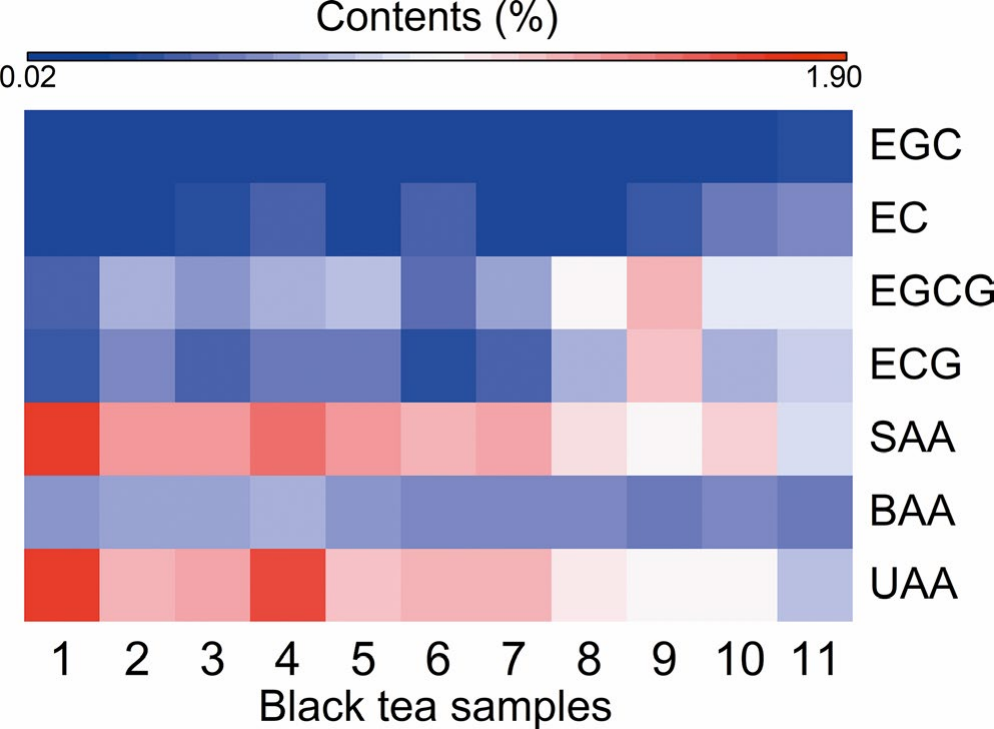
Heat map of catechin monomers [(‐)‐Epigallocatechin (EGC), (‐)‐epicatechin (EC), (‐)‐epigallocatechin gallate (EGCG), and (‐)‐epicatechin gallate (ECG)], sweet amino acids (SAA), bitter amino acids (BAA), and umami amino acids (UAA) contents in tea samples from a single spring season. Contents of components are illustrated on a red (high) to blue (low) scale

Hence, we determined the catechins and free amino acids contents (Figure 4). The contents of EGCG, ECG, EC, and EGC were higher than other catechins, which is consistent with observations from previous studies (Kim, Choi, & Park, 2015; Narukawa et al., 2011; Saklar, Ertas, Ozdemir, & Karadeniz, 2015). The EGCG and ECG contents increased significantly over time, whereas the EC and EGC contents showed no obvious change (Figure 4). Table S2 shows that the variation coefficient for the amino acid monomers were more than 15%, indicating a great difference in the content of amino acid components in black tea samples throughout spring. Furthermore, the theanine, asp, ser, glu, gly, his, arg, pro, phe, and thr contents were relatively higher than other amino acids. Specifically, theanine originally accounted for more than 50% of the total amino acids content was found to decrease over time, and this variation was consistent with previous report (Chatterjee, Chatterjee, & Bandyopadhyay, 2016). Theanine contributes to the sweet taste (Ekborg‐Ott, Taylor, & Armstrong, 1997; Yu, & Yang, 2020) as well as the umami (Juneja, Chu, Okubo, Nagato, & Yokogoshi, 1999; Narukawa, Toda, Nakagita, Hayashi, & Misaka, 2014) taste of tea infusions. Thus, free amino acids can be divided into sweet amino acids (gly, ser, ala, pro, thr, met, and theanine), bitter amino acids (arg, his, ile, leu, phe, lys, tyr, and val), and umami amino acids (asp, glu, and theanine) according to the different taste characteristics of amino acids (Scharbert & Hofmann, 2005; Zhang et al., 2017), and the contents of these amino acids were investigated (Figure 4). We observed a decrease in the contents of sweet amino acids and umami amino acids over time, whereas a change in the content of bitter amino acids was not obvious throughout the spring. Together, these results suggested that the ester catechins, sweet amino acids, and umami amino acids were critical for the formation of tea taste quality.

**Figure 5:**
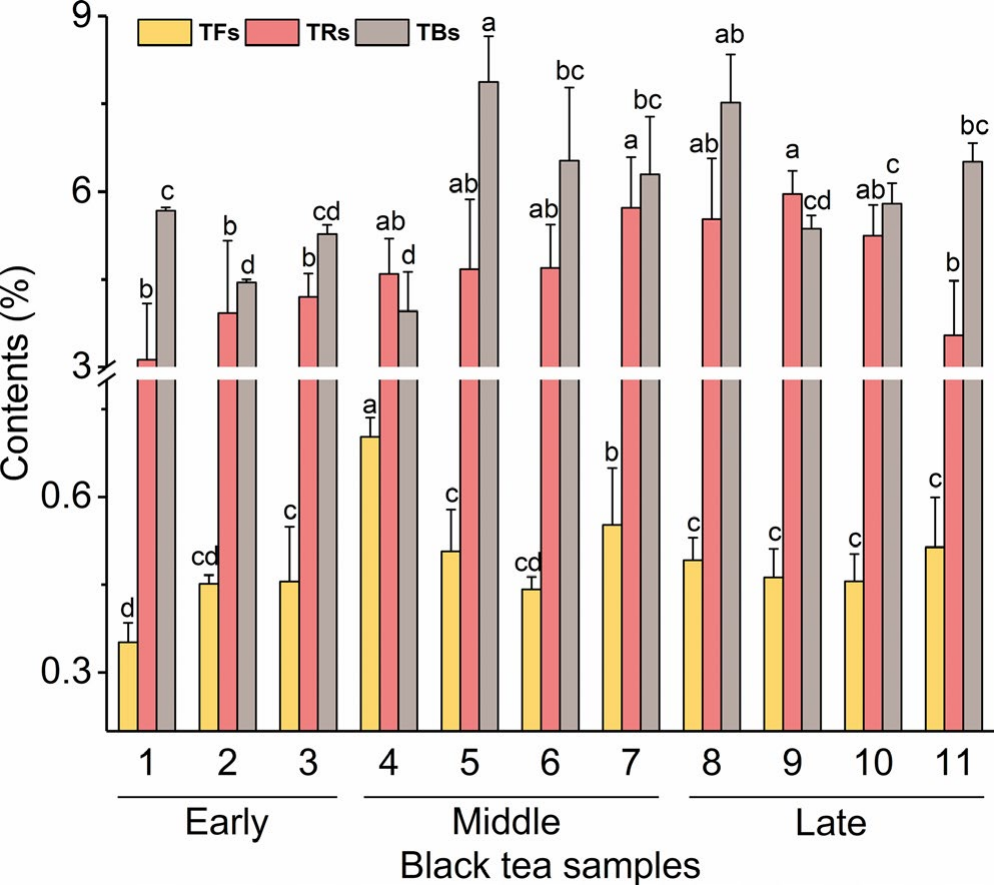
Variations of theaflavins (TFs) content, thearubigins (TRs) content, and theabrownins (TBs) content in black tea samples manufactured during a single spring season. Different lowercase letters in the figure indicate significant (*p* < .05) differences in the content of compounds

TFs, TRs, and TBs are formed by enzymatic oxidation of catechins and gallates during the fermentation/oxidation stage of tea processing, playing an important role in the taste quality by affecting the astringency and intensity of black tea infusion (Bhuyan, Borah, Sabhapondit, Gogoi, & Bhattacharyya, 2015; Scharbert, Jezussek, & Hofmann, 2004b). Our results showed that the TFs content changed significantly in the early spring and mid‐spring samples, while the TRs and TBs content exhibited irregular fluctuation in all samples (Figure 5).

### 3.4 Chemical compounds correlated with taste quality of black tea

**Figure 6:**
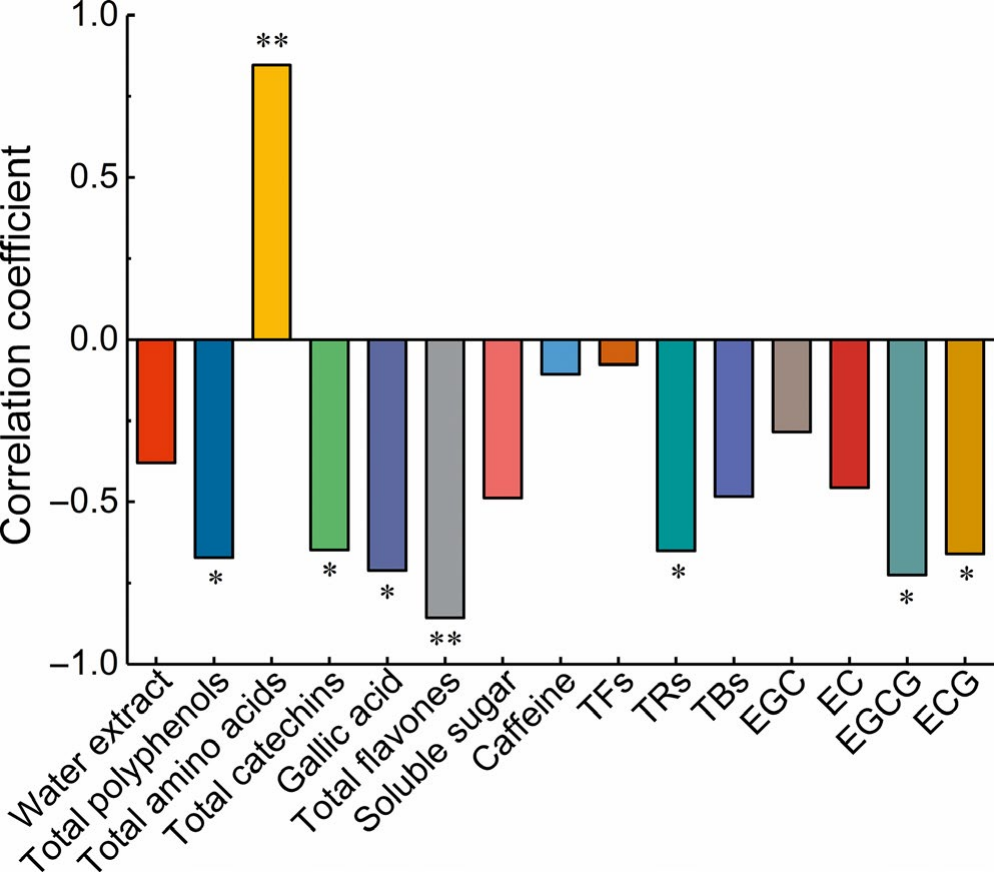
Correlation coefficient of the taste average score and chemical compounds. (*) *p* < .05; (**) *p* < .01

To further investigate the relationship between taste compounds and taste quality, we conducted a Pearson's correlation coefficient analysis. The TAA content in the infusions was significantly positively correlated with the taste quality score (*p* < .01, r = 0.846). In addition, the content of total flavones was significantly inversely correlated with the taste score (*p* < .01, r = ‐ 0.858). Moreover, the ECG, EGCG, TC, TP, GA, and TR contents were also significantly inversely correlated with the taste quality score (*p* < .05). Surprisingly, the correlations between the contents of WE, SS, CAF, EC, EGC, TFs, TBs and taste average score were not significant (Figure 6). Taken together, these results suggested that TAA, and total flavones retained greater influence on the taste quality in black tea, followed by ECG, EGCG, GA, TC, TP, and TRs.

## 4 DISCUSSION

In this study, we found that the taste quality of HJC Congou black tea manufactured in the early spring was better than that in the late spring. The contents of TAA and total flavones, but not the bitter‐tasting CAF, had a significant correlation with the variation in taste quality for HJC Congou black tea manufactured during a single spring season.

HJC is a special tea variety, and the leaves of which possess high amounts of free amino acids and have a high leaf yield (Li et al., 2017) in Hunan, China. In our earlier studies, the quality of green tea made from HJC leaves was superior in the early spring, reaching a TAA content as high as 7.47%. HJC leaves also exhibited an advantage in the manufacturing of black tea during the spring season. In this study, we found that the taste quality of HJC Congou black tea manufactured in the early spring was significantly better than that manufactured in the late spring via sensory evaluation (Figure 1). Although sensory evaluation is susceptible to subjective factors, most studies referenced this method in evaluating tea quality. Specifically, Zhang et al. (2017) investigated the color, aroma, and taste quality of Fuding white tea infusion based on sensory evaluation. Xu, Zhang, et al. (2018b) analyzed the taste quality of green tea infusions using the results of a sensory evaluation team with nine panelists.

In order to verify the sensory evaluation results, we further investigated the taste quality using electronic tongue taste analysis. The electronic tongue is widely used in tea taste assessment and evaluation for its high reproducibility, sensitivity, and reliability (Saha, Ghorai, Tudu, Bandyopadhyay, & Bhattacharyya, 2018; Zou, Xiao, Wang, & Zhang, 2018). By performing PCA (Figure 2a) and cluster analysis (Figure 2b) on the data from the electronic tongue sensor, we obtained results consistent with the sensory evaluation. The taste quality of black tea manufactured in spring can be clearly discriminated. Interestingly, the taste quality of black tea manufactured before and after April 4 was separated by PC1, implying April 4 may be the critical time point on which taste quality begins to vary. This result was in agreement with previous studies reporting that the taste quality of Xihulongjing green tea can be discriminated before and after the Qingming festival using an electronic tongue (Xiao & Wang, 2009). We hypothesized that the critical time point for taste variation may be attributed to the difference in taste compounds present in teas manufactured during different periods of the spring season.

Based on these results, the variation in the primary taste compounds was analyzed. Many studies have reported that tea compound content changes in different seasons (Chatterjee et al., 2016; Fujimori, Suzuki, & Ashihara, 1991; Turkmen & Velioglu, 2007). Wang, Wei, et al. (2011b) evaluated the effects of seasonal climate change on catechins contents and observed that the EGC, EC, ECG, and EGCG levels increased; however, (+)‐catechin decreased with increasing daily average temperature. We observed a similar increasing trend in the TP, TC, ECG, and EGCG contents over time as spring progressed (Figures 3 and 4). Although the EC and EGC contents were quite low in comparison with the ECG and EGCG contents, a significant increase was also observed from early spring to the late spring. Furthermore, the average catechin content in leaves plucked during the summer was more abundant than that observed in the spring (Xu et al., 2012), whereas the PPO and POD activities were lower in fresh leaves plucked in summer than in spring (Jiang et al., 2018). Variation in the level of catechins in our study may due to the higher catechins contents and simultaneous low enzymatic activity in the late spring.

Specific flavones and their glycosides have been reported to increase in the late spring for the reason of higher temperature and light intensity (Zhang, Shi, Ma, Yi, & Ruan, 2014). The contents of quercetin and their glycosides increased while the contents of myricetin and their glycosides decreased from the early to late spring (Liu et al., 2016). Moreover, the variable trends in flavones and their glycosides levels depend on the aglycone species in different seasons. The apigenin‐C‐glycosides content and quercetin‐O‐glycosides content were higher in summer tea samples than in spring tea samples, while the kaempferol‐O‐glycosides had the lowest content in summer tea samples (Dai et al., 2015). In our study, the total flavones content exhibited a similar temperature‐dependent increasing trend as described above from early spring (lower temperature) tea samples to late spring tea samples, implying a greater level of increase than decrease in flavones content over a single spring season.

Further, Jiang et al., (2018) observed that the production of TFs was higher in the early spring than in the late spring, whereas the TRs content exhibited a reverse “U”‐shaped variation in spring. We also found a reverse “U”‐shaped variation in TRs content, whereas the contents of TFs and TBs showed irregular fluctuations (Figure 5). The difference in variation of TFs and TBs contents may due to the different varieties utilized during the production of Congou black tea.

Many studies have reported a relationship between taste quality and taste compounds. Flavonoids, including catechins, flavones, and their glycosides, are believed to be main taste components in black tea infusion. The catechins in tea polyphenols, especially ester catechins, are the main contributors to the astringent and bitter taste of tea infusion (Chen, Zhao, Guo, & Wang, 2010; Narukawa, Kimata, Noga, & Watanabe, 2010; Yu, Yeo, Low, & Zhou, 2014). Flavones, primarily existing in glycoside forms of kaempferol, quercetin, and myricetin in tea, not only have important impact on the velvety astringent taste of black tea, but also contribute to the bitter taste by amplifying the bitterness of CAF (Scharbert & Hofmann, 2005). Additionally, free amino acids asp, glu, and theanine have been reported to highly correlate with umami taste and enhance the umami intensity of tea infusions (Kaneko, Kumazawa, Masuda, Henze, & Hofmann, 2006). Pearson's correlation analysis in this study showed similar results with those above such that the changes in TAA, and total flavones were highly significantly (*p* < .01) correlated with taste quality score (Figure 6). Interestingly, the content of total catechins significantly (*p* < .05) correlated with taste quality score, suggesting less importance of catechins in the formation of black tea taste compared with flavones. This result corresponded well to previous report that flavones and their glycosides were the main contributors to black tea taste for the sake of extremely low threshold concentrations (Scharbert, Holzmann, & Hofmann, 2004a).

Inconsistent results have reported that free amino acids may not be important for the taste formation since the concentration of each amino acid was much lower than their recognized threshold concentration in Darjeeling black tea infusions (Scharbert & Hofmann, 2005) and white tea infusions (Yang et al., 2018). Herein, taste average scores were significantly positively correlated with the total contents of sweet or umami amino acids (Figure 6), which may be due to the fact that we investigated the correlation between taste average scores and the total contents of sweet or umami amino acids, instead of analyzing the dose‐over‐threshold factor (Dot) of a single amino acid. In fact, free amino acids play an important role in not only presenting sweet and umami taste, but also inhibiting the bitter taste caused by CAF and catechins (Takeo, 1981). Thus, the interaction of amino acids and other compounds on taste characteristics requires further analysis.

CAF was identified as the most important bitter‐tasting compound in tea infusions, while TFs, TRs, and TBs generated during tea fermentation are believed to impart the mouth‐coating, astringent, and long‐lasting oral sensation of black tea infusions (Scharbert, Holzmann, et al., 2004a). However, no significant differences were observed when correlating average taste score with CAF, TFs, and TBs contents, save for TRs. A possible explanation is the irregular fluctuation of these three compounds' content in black tea manufactured throughout the spring season.

The taste quality of Congou black tea may be influenced by the variety, region, and manufacturing process. In this study, tea leaves of the HJC variety plucked from the same garden were selected as raw materials and manufactured into Congou black tea through the same process in order to investigate the seasonal effect on taste quality. Based on the results of sensory evaluation, electronic tongue analysis, main taste compounds analysis, and correlation analysis, we found that the taste quality of HJC black tea in the early spring was better than that in the late spring and that the critical time point at which taste quality variations begin may be April 4. Furthermore, TAA and total flavones were primarily responsible for the seasonal variations in taste quality of black tea. However, this study only analyzed the variation of highly abundant taste compounds during a single spring season and failed to investigate the flavor characteristics in other potential taste compounds. It is, therefore, necessary to further analyze the taste quality with comprehensive metabolism profiling.

In conclusion, the taste quality of HJC black tea manufactured in a single spring season was described and the key compounds of the taste quality variation were investigated in this study. Considering that taste quality is a key factor for tea quality, our observations may serve to provide new insights for the processing of Congou black tea in the spring season.

## CONFLICT OF INTEREST

The authors declare that they have no conflicts of interest.

## ETHICAL APPROVAL

This study does not involve any human or animal testing. Written informed consent was obtained from all study participants.
