# Supplementary material for: Distinct variation in taste quality of Congou black tea during a single spring season
Source: Food Sci Nutr. 2020 Feb 18;8(4):1848–56. doi: 10.1002/fsn3.1467 (PMC7174197; doi:10.1002/fsn3.1467)
Supplement: Supplementary file 1 [file FSN3-8-1848-s001.pdf]

Supporting Information

Figure S1 Relative standard deviation of the sensors from Congou black tea infusions. SRS, SWS, BRS, STS, UMS, GPS, and SPS, representing sour, sweet, bitter, salty, umami, and two combination tastes, respectively.

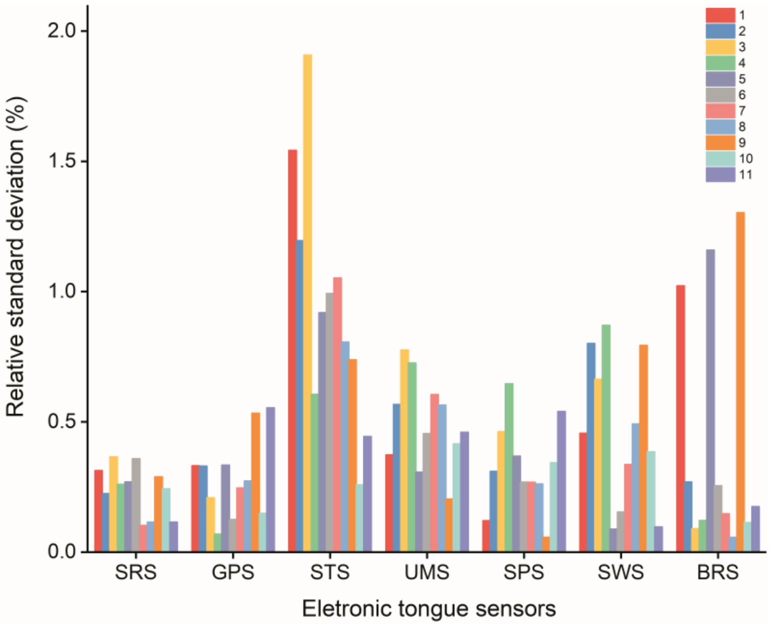

Table S1 Eigenvalues and variance of principal components

Principal components number	Eigenvalue	Percentage of Variance (%)	Cumulative (%)
1	18.23	77.86	77.86
2	4.33	18.48	96.34
3	0.64	2.73	99.07
4	0.13	0.57	99.64
5	0.07	0.29	99.93
6	0.02	0.05	99.98
7	0.01	0.02	100

Table S2 Amino acid monomer mean contents (%) of black tea samples (BTS)

BTS	Asp	Ser	Glu	Gly	His	Arg	Thr	Ala	Pro	Cys	Tyr	Val	Met	Lys	Ile	Ike	Phe	Theanine
1	0.119	0.143	0.280	0.003	0.152	0.196	0.038	0.036	0.160	0.000	0.024	0.017	0.002	0.036	0.024	0.045	0.042	1.527
2	0.090	0.273	0.282	0.006	0.081	0.118	0.050	0.043	0.136	0.003	0.038	0.050	0.004	0.068	0.055	0.082	0.107	0.965
3	0.120	0.241	0.272	0.005	0.102	0.126	0.052	0.042	0.124	0.001	0.045	0.044	0.003	0.059	0.048	0.068	0.096	0.988
4	0.110	0.139	0.434	0.004	0.157	0.243	0.042	0.041	0.122	0.001	0.034	0.023	0.004	0.035	0.034	0.062	0.056	1.262
5	0.106	0.172	0.167	0.004	0.086	0.129	0.049	0.049	0.146	0.001	0.061	0.034	0.004	0.059	0.044	0.071	0.097	0.998
6	0.128	0.128	0.185	0.003	0.077	0.060	0.039	0.030	0.109	0.001	0.049	0.039	0.003	0.049	0.038	0.060	0.101	0.997
7	0.122	0.148	0.178	0.007	0.108	0.063	0.043	0.031	0.103	0.001	0.042	0.026	0.005	0.046	0.033	0.055	0.097	1.040
8	0.120	0.162	0.184	0.002	0.065	0.049	0.043	0.037	0.106	0.003	0.036	0.038	0.005	0.048	0.042	0.055	0.111	0.790
9	0.172	0.157	0.184	0.005	0.047	0.032	0.050	0.043	0.120	0.002	0.058	0.042	0.005	0.054	0.044	0.062	0.110	0.643
10	0.090	0.200	0.151	0.006	0.049	0.059	0.036	0.032	0.099	0.004	0.039	0.046	0.003	0.049	0.041	0.053	0.116	0.795
11	0.096	0.222	0.120	0.003	0.046	0.043	0.023	0.031	0.068	0.001	0.036	0.046	0.002	0.035	0.032	0.039	0.105	0.486
SD	0.023	0.047	0.089	0.001	0.039	0.068	0.008	0.006	0.025	0.001	0.011	0.011	0.001	0.011	0.009	0.012	0.023	0.283
CV%	19.74	26.12	40.08	32.18	44.15	67.11	19.67	16.61	21.15	72.48	25.72	28.58	32.77	22.40	21.49	20.28	24.80	29.70
